# Supplementary material for: Accuracy of the Low-Dose ACTH Stimulation Test for Adrenal Insufficiency Diagnosis: A Re-Assessment of the Cut-Off Value
Source: J Clin Med. 2019 Jun 5;8(6):806. doi: 10.3390/jcm8060806 (PMC6616948; doi:10.3390/jcm8060806)
Supplement: Supplementary File 1 [file jcm-08-00806-s001.pdf]

N.	Signs and/or symptoms, comorbidities	Suspicion of primary or central AI	Follow-up (months)
1	Asthenia, hypopituitarism, previous pituitary macroadenoma	Central	>24 months
2	Asthenia, chronic autoimmune thyroiditis	Primary	12 months
3	Asthenia, weight loss	Primary	>24 months
4	Lipotimia	Primary	>24 months
5	Lipotimia, asthenia	Primary	>24 months
6	Lipotimia, vertigo, asthenia	Primary	13 months
7	Vertigo, headhache, hypotension, mestrual disorders	Uncertain	24 months
8	Asthenia	Uncertain	12 months
9	Chronic autoimmune thyroiditis, hypotiroidism, asthenia	Primary	>24 months
10	Asthenia, familiar history of autoimmune polyglandular syndrome	Primary	12 months
11	Chronic autoimmune thyroiditis, vitiligine, Asthenia	Primary	18 months
12	Asthenia, chronic autoimmune thyroiditis	Primary	>24 months
13	Hypopituitarism	Central	>24 months
14	Migraine headache, chronic autoimmune thyroiditis	Primary	>24 months
15	Asthenia	Uncertain	15 months
16	Chronic autoimmune thyroiditis, autoimmune gastritis, adrenal cortex autoantibodies posititvy	Primary	>24 months
17	Chronic autoimmune thyroiditis, asthenia, vitiligo	Primary	>24 months
18	Weight loss	Primary	20 months
19	Asthenia	Uncertain	12 months
20	Asthenia	Uncertain	>24 months
21	Hypogonadism, asthenia	Uncertain	>24 months
22	Asthenia	Uncertain	>24 months
23	Hypogonadism	Primary	>24 months
24	Chronic autoimmune thyroiditis, diabetes mellitus, Basedow's disease	Primary	18 months
25	Lipotimia	Uncertain	>24 months
26	Lipotimia, asthenia	Uncertain	>24 months
27	Asthenia, weight loss	Primary	>24 months
28	Chronic autoimmune thyroiditis	Primary	>24 months
29	Asthenia	Primary	12 months
30	Prolactinoma, vitiligo, coeliac disease	Uncertain	>24 months
31	Hyperprolactinemia	Central	12 months
32	Asthenia	Primary	>24 months
33	Asthenia	Uncertain	12 months
34	Hypogonadism, asthenia	Primary	12 months
35	Hypogonadism, lipotimia	Primary	18 months
36	Insomnia, tachycardia	Primary	>24 months
37	Asthenia	Primary	>24 months
38	Asthenia, lipotimia	Uncertain	13 months

39	Chronic autoimmune thyroiditis, insomnia	Primary	>24 months
40	Asthenia	Uncertain	>24 months
41	Hypogonadism, thalassemia, hypotiroidism	Central	>24 months
42	Asthenia	Primary	15 months
43	Asthenia	Uncertain	>24 months
44	Lipotimia	Uncertain	18 months
45	Chronic autoimmune thyroiditis, headache, asthenia, adrenal cortex autoantibodies positivity	Primary	>24 months
46	Hypotension, lipotimia	Primary	>24 months
47	Asthenia	Primary	12 months
48	Chronic autoimmune thyroiditis, asthenia	Uncertain	13 months
49	Hypopituitarism	Central	>24 months
50	Chronic autoimmune thyroiditis, asthenia	Uncertain	12 months
51	Asthenia, weight loss	Uncertain	13 months
52	Chronic autoimmune thyroiditis, asthenia	Primary	>24 months >24 months
53	Hypogonadism, asthenia	Uncertain	18 months
54	Insomnia, hypotension, diarrhea	Primary	12 months
55	Asthenia	Uncertain	12 months
56	Hypopituitarism, pituitary macroadenoma,	Central	>24 months
57	Chronic autoimmune thyroiditis, coeliac disease, arthritis	Primary	>24 months
58	Chronic autoimmune thyroiditis, coeliac disease	Primary	12 months
59	Hypogonadism	Uncertain	>24 months
60	Asthenia	Uncertain	18 months
61	Asthenia	Uncertain	18 months
62	Asthenia	Primary	12 months
63	Lipotimia	Uncertain	12 months
64	Thalassemia, hypogonadism, GH deficit	Central	>24 months
65	Chronic autoimmune thyroiditis	Primary	12 months
66	Asthenia	Primary	12 months
67	Chronic autoimmune thyroiditis, asthenia, pituitary adenoma	Central	15 months
68	Asthenia, nausea	Primary	12 months
69	Asthenia	Primary	18 months
70	Hyponatremia	Primary	>24 months
71	Hypoglicemy, vertigo, hypotension	Primary	Dropped out
72	Chronic autoimmune thyroiditis, asthenia	Primary	>24 months
73	Asthenia	Primary	>24 months
74	Lipotimia	Uncertain	18 months
75	Chronic autoimmune thyroiditis	Uncertain	12 months
76	Chronic autoimmune thyroiditis, ipotiroidismo	Primary	>24 months
77	Chronic autoimmune thyroiditis	Primary	>24 months
78	Previuos Sheehan's syndrome	Central	12 months
79	Asthenia	Uncertain	12 months
80	Diabetes mellitus	Uncertain	>24 months

81	Asthenia	Uncertain	15 months
82	Asthenia	Primary	12 months
83	Headache, asthenia, defluvium	Uncertain	12 months
84	Asthenia	Uncertain	18 months
85	Chronic autoimmune thyroiditis, Sjogren's syndrome	Primary	>24 months
86	Asthenia	Primary	>24 months
87	Asthenia, head trauma	Central	12 months
88	Chronic autoimmune thyroiditis, hypotension	Primary	12 months
89	Asthenia	Primary	>24 months
90	Asthenia, hypogonadism, hypotioridism, previous pituitary adenoma	Central	>24 months
91	Pituitary adenoma	Central	18 months
92	Asthenia	Uncertain	12 months
93	Asthenia	Central	12 months
94	Hypopituitarism	Central	>24 months
95	Asthenia	Central	>24 months
96	Asthenia, headache	Primary	>24 months
97	Asthenia	Primary	12 months
98	Thalassemia, hypogonadism	Central	12 months
99	Asthenia	Uncertain	18 months
100	Asthenia	Primary	>24 months
101	Asthenia	Uncertain	12 months
102	Hypotension	Uncertain	12 months
103	Hypogonadism	Central	12 months

Table. Principal characteristics leading to the suspect of AI and duration of follow-up after testing. Patients with AI are highlighted in yellow.
